# Isolated Supinator Muscle Tear With Associated Undisplaced Radial Neck Fracture Following Trauma: A Rare Case

**DOI:** 10.7759/cureus.94082

**Published:** 2025-10-07

**Authors:** Vishakh Karicheri Gangadharan, Vishwas Kadambila, Rakesh Sera, Sushil KP, John Joe Jacob

**Affiliations:** 1 Orthopaedics, Kanachur Institute of Medical Sciences, Mangalore, IND

**Keywords:** conservative management, elbow injury, forearm supination, high grade muscle tear, supinator muscle

## Abstract

Isolated injuries to the supinator muscle are exceedingly uncommon, with most reports documenting its involvement alongside tendon or ligament injuries rather than as a distinct lesion. We present the case of a 42-year-old male who sustained a high-grade supinator muscle tear associated with an undisplaced radial neck fracture following blunt trauma. The patient was diagnosed using magnetic resonance imaging (MRI) after initial clinical suspicion. Conservative management consisting of short-term immobilization and progressive physiotherapy led to near-complete recovery by 12 weeks, with sustained functional results at 18 months. This report emphasizes the importance of considering isolated muscular injuries in the differential diagnosis of post-traumatic elbow pain and the role of MRI in excluding concomitant pathology.

## Introduction

Elbow injuries are most frequently associated with osseous, ligamentous, or tendon-related pathology, whereas isolated muscular injuries around the elbow are rarely described. Among these, supinator muscle tears are particularly unusual. The supinator is a deep forearm muscle originating from the lateral epicondyle, radial collateral ligament, annular ligament, and supinator crest of the ulna, inserting onto the proximal third of the radius. Its primary function is to facilitate supination of the forearm, especially in elbow extension when the biceps brachii is less effective.

Because of its protected anatomical position beneath the extensor muscle mass and adjacent ligamentous structures, traumatic injuries to the supinator are rare. When they occur, they often coexist with other structural lesions, such as distal biceps tendon rupture, ulnar collateral ligament (UCL) tears, or posterolateral rotatory instability. For example, Nayyar et al. described a case of supinator tear with distal biceps tendon rupture in a middle-aged woman [1]. Similarly, avulsion of the supinator crest has been reported in association with posterolateral elbow instability [2,3].

To our knowledge, very few cases of high-grade supinator tears confirmed radiologically have been described, and almost all were associated with additional ligamentous or osseous pathology. Here, we present a case of traumatic high-grade supinator tear with an associated undisplaced radial neck fracture, diagnosed on MRI and successfully treated conservatively.

## Case presentation

A 42-year-old right-hand-dominant male sustained an injury to his right elbow after a road traffic accident in which the elbow struck a car door directly. He reported immediate pain, swelling, and difficulty rotating the forearm.

Clinical examination

Swelling was noted over the volar and lateral aspects of the proximal forearm. Mild tenderness was elicited in this region. Elbow flexion and extension were preserved, but forearm rotations were markedly restricted, with supination being particularly painful and limited. Neurovascular examination of the limb was normal, with intact radial and ulnar pulses and preserved sensory-motor function. Based on the mechanism of injury and clinical findings, differentials included a radial head or neck fracture, distal biceps tendon injury, or lateral collateral ligament injury. 

Imaging

**Figure 1:**
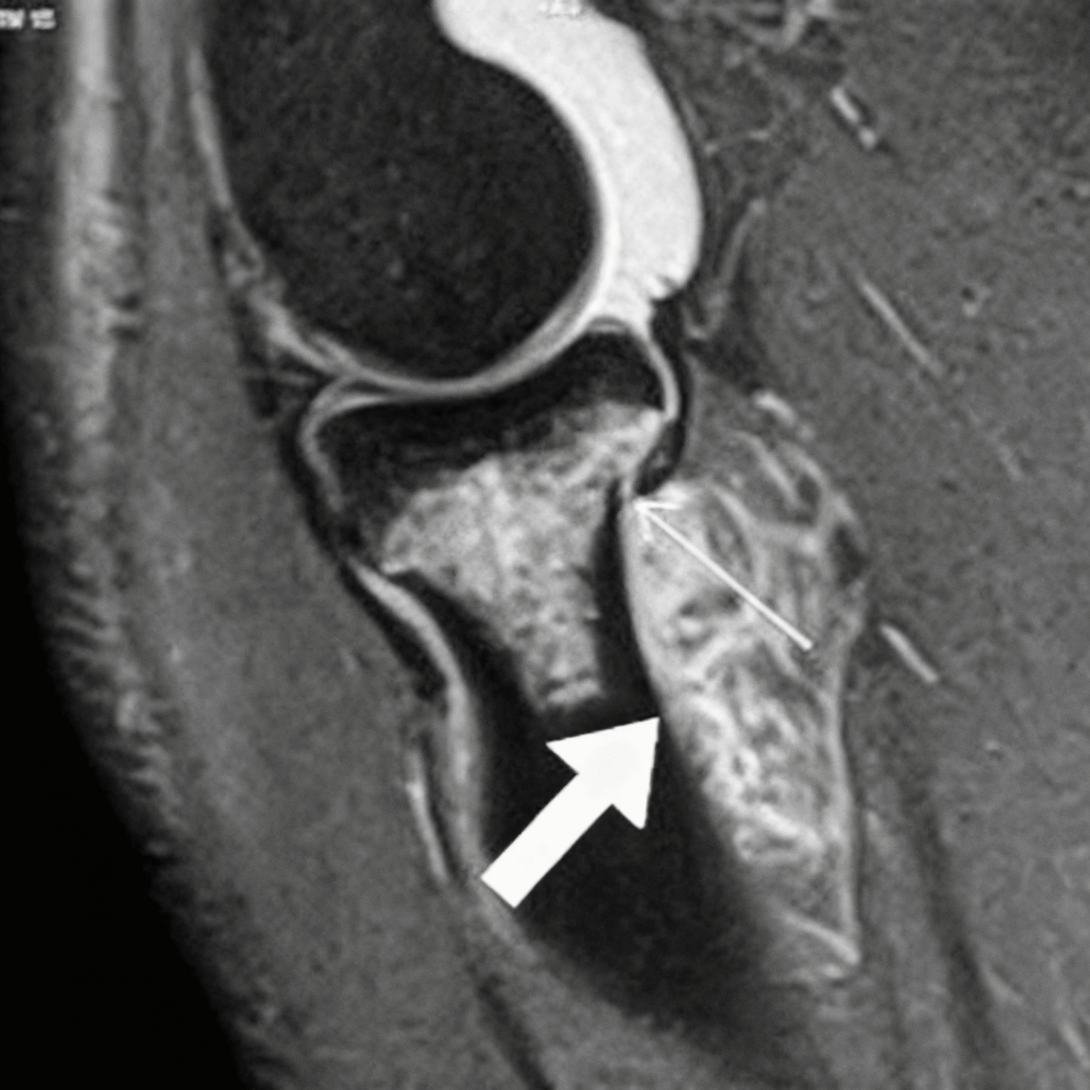
MRI image The thick arrow shows the supinator muscle high-grade tear; the thin arrow shows an undisplaced radial neck fracture.

Plain radiographs were inconclusive, showing no clear fracture line. MRI of the right elbow revealed diffuse edema and high signal intensity within the supinator muscle, consistent with a high-grade tear involving more than 50% of the muscle belly (approximate length of 38 mm), along with moderate joint effusion. An undisplaced fracture of the radial neck with associated marrow edema was also noted (Figure 1).

The distal biceps tendon, radial and ulnar collateral ligaments, and neurovascular structures were intact. These findings confirmed a high-grade supinator tear associated with an undisplaced radial neck fracture, without ligamentous instability.

Management

The patient was treated conservatively. He was immobilized in a sling for two weeks for pain reduction and fracture stabilization, after which supervised physiotherapy was initiated. Rehabilitation focused on gradual restoration of the range of motion and progressive strengthening, with special emphasis on controlled supination exercises. These began as passive-assisted movements and progressed to active and resisted supination.

At six weeks, the patient reported marked pain relief with near-full restoration of supination and pronation. By 12 weeks, he was able to perform all activities of daily living without restriction. At the 18-month follow-up, he remained asymptomatic with full functional recovery and no recurrence of symptoms.

## Discussion

Supinator tears are exceptionally rare, partly due to the muscle’s deep location and protection from direct trauma. When they occur, they are usually associated with more obvious injuries. Nayyar et al. reported a distal biceps tendon rupture with concomitant supinator tear [1]. Avulsion fractures involving the supinator crest have been recognized as markers of posterolateral instability, as highlighted by Broekhuis et al. [2] and Schmidt-Horlohé et al. [3]. Our case is distinct because the supinator tear occurred alongside only an undisplaced radial neck fracture, without tendon or ligament injury. This emphasizes that high-grade supinator tears can occur as primary muscular injuries in blunt trauma and should not be overlooked.

Clinically, an isolated supinator injury is difficult to diagnose due to overlapping symptoms with more common injuries. Key clues include pain and swelling over the proximal volar forearm, selective loss of supination despite preserved elbow flexion-extension, and relatively normal radiographs. In our patient, suspicion for radial head fracture or distal biceps tendon tear prompted MRI. The scan confirmed the muscle tear and excluded other injuries. MRI also enabled the detection of the subtle radial neck fracture. Demino et al. similarly demonstrated MRI’s value in identifying overlooked soft-tissue and osseous injuries following elbow dislocation [4].

Management of muscle injuries typically favors conservative strategies. Järvinen et al. outlined that intramuscular tears generally heal well with rest, protection, and structured rehabilitation [5]. Our treatment strategy aligned with these principles, emphasizing short-term immobilization followed by graded mobilization. Rehabilitation after elbow injury must balance protection of healing tissues with prevention of stiffness, and in this patient, careful progression allowed complete recovery within 12 weeks.

Surgical intervention for supinator injuries is rarely necessary, generally reserved for cases with instability or significant bony avulsion. O’Driscoll et al. emphasized the surgical relevance of posterolateral instability but did not advocate surgery for isolated muscular injuries without instability [6]. This case highlights several lessons: supinator tears should be suspected when patients present with selective loss of supination despite preserved elbow motion after trauma; MRI is the diagnostic modality of choice; conservative treatment is highly effective; and associated subtle bony injuries, such as undisplaced radial neck fractures, must be carefully assessed.

## Conclusions

Isolated supinator muscle tears are exceedingly rare and may mimic more common elbow injuries. This case demonstrates that such injuries can occur in association with undisplaced radial neck fractures without ligamentous or tendon involvement. MRI is invaluable for accurate diagnosis, enabling conservative management and preventing unnecessary surgical intervention. With appropriate nonoperative treatment, excellent recovery can be achieved within 12 weeks and maintained long term. This report adds to the limited literature on supinator injuries and emphasizes the importance of considering isolated muscular tears in patients with post-traumatic elbow pain and restricted supination.
